# Lactation during cholestasis: Role of ABC proteins in bile acid traffic across the mammary gland

**DOI:** 10.1038/s41598-017-06315-8

**Published:** 2017-08-07

**Authors:** Alba M. G. Blazquez, Rocio I. R. Macias, Candela Cives-Losada, Alberto de la Iglesia, Jose J. G. Marin, Maria J. Monte

**Affiliations:** 1 Experimental Hepatology and Drug Targeting (HEVEFARM), IBSAL, University of Salamanca, Salamanca, Spain; 2 Center for the Study of Liver and Gastrointestinal Diseases (CIBERehd), Carlos III National Institute of Health, Madrid, Spain

## Abstract

Transporters involved in bile acid (BA) handling by the mammary gland are poorly understood. Here we have investigated the role of ABC proteins in blood-milk BA traffic and its sensitivity to maternal cholestasis. BA concentrations in rat and mouse serum were higher than in milk. BA profiles in both fluids were also different. In mammary gland, mRNA levels of ABC pumps transporting BAs were high for Bcrp, less abundant for Mrp1, Mrp3 and Mrp4 and negligible for Bsep and Mrp2. Milk BA concentrations were lower in *Abcg2* ^−/−^ than in wild-type mice. Taurocholate administration (5 µmol, i.p.) increased 20-fold BA concentrations in serum, but only moderately in milk, even in *Abcg2* ^−/−^ mice. Bile duct ligation (BDL) in pregnant rats markedly increased serum BA concentrations, which was not proportionally reflected in milk. In rat mammary tissue, Mrp4 was up-regulated by BDL. Serum BA levels were 2-fold higher in 10-day-old neonates of the BDL group, whereas their body weight was lower. The exchange of breastfeeding mothers immediately after birth reverted the situation without changes in endogenous BA synthesis. In conclusion, Bcrp is involved in BA secretion into milk, whereas Mrp4 participates in a blood-milk barrier that protects neonates from maternal hypercholanemia during breastfeeding.

## Introduction

Intrahepatic cholestasis of pregnancy (ICP) is the most frequent pregnancy-specific liver disease^1^. This reversible form of cholestasis is characterized by pruritus and elevated bile acid (BA) concentrations in maternal serum and is accompanied by an increased risk of intrauterine and neonatal complications^2–5^. Although many efforts have been taken to elucidate the etiopathogenesis of this disease^6, 7^ and to establish an appropriate clinical management of these pregnancies^8, 9^, the influence of ICP on the long-term development and health status of the offspring remains poorly understood. In animal models, a transient impairment of hepatobiliary function in young animals born from mothers with hypercholanemia during pregnancy has been reported^10, 11^. In both mice and humans, cholestasis during pregnancy, in the absence of other maternal alterations, has recently been associated with programmed metabolic disease in the offspring^12^. Despite the suggested etiological role of the intrauterine exposure to high levels of BAs, little is known about the effect of changes in BA supply to the neonates during early lactation. BAs are present in human breast milk^13, 14^, although their biological significance in this fluid is only partly understood. In the colostrum of women with ICP, BA concentrations are higher than normal (≈1 µM)^15^, but the magnitude of the difference is controversial because it varied from 2-fold in a group of 7 patients to ≈50-fold in the other 2 patients included in the same study^15^. Although the signs and symptoms of ICP resolve within 2–4 weeks after delivery^16^, it is not known whether high levels of BAs in milk persist in ICP patients during early lactation and how this might affect neonatal BA homeostasis. This is important, for instance, because the BA composition and concentration of breast milk may influence the microbiota present in milk and thereby affect the development of gut microbiota in the lactating newborn^17^.

In this context, it is important to elucidate the underlying mechanisms accounting for BA traffic between blood and milk. In addition to members of the solute carrier (SLC) family of transporters, still poorly identified in mammary gland, several members of the ATP-binding cassette (ABC) superfamily of membrane transporters may be involved in the secretory/barrier function of mammary epithelium. Thus, MRP3 (gene symbol *ABCC3*) and MRP4 (*ABCC4*) are expressed on the basolateral membrane of hepatocytes. However, in response to obstructive cholestasis or BA feeding in mice only Mrp4 undergoes adaptive up-regulation. Moreover, although Mrp3 is up-regulated in *Mrp4* ^−/−^ mice, this does not compensate for the loss of Mrp4-induced protection during cholestasis^18^. At the apical pole of the epithelial plasma membrane, the breast cancer resistance protein (BCRP, *ABCG2*), behaves as an efflux pump that exhibits a broad range of substrate specificity and hence is able to transport a variety of endogenous compounds and xenobiotics out of the cells^19–22^. Regarding the physiological role of BCRP in the mammary gland during pregnancy and lactation, this transporter is up-regulated in alveolar epithelial cells, where it has been associated with the transfer of different endogenous compounds into milk, such as vitamin B_2_ (riboflavin) and possibly other types of vitamins^23^. BCRP has recently been shown to play a key role in BA transport across the placental trophoblast^24^, where this carrier is also highly expressed^25, 26^.

Te goal of the present study was to investigate the role of ABC pumps in the traffic of BAs across the mammary gland and the effect of maternal hypercholanemia on BA secretion into milk. To achieve this aim, two animal models were used: i) lactating rats with or without obstructive cholestasis during pregnancy, to mimic maternal hypercholanemia present in ICP patients, and ii) lactating wild-type and Bcrp knockout (*Abcg2* ^−/−^) mice, for assessing the overall contribution of Bcrp to BA secretion into milk.

## Results

### Presence of BAs in rat and mouse milk

**Figure 1:**
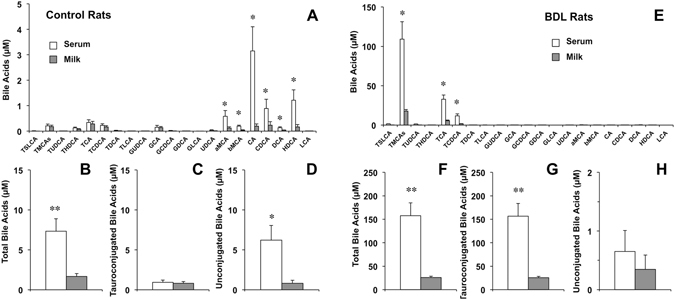
Bile acid concentrations as determined by HPLC-MS/MS in paired samples of serum and milk collected on day 10 after delivery from healthy lactating rats (n = 6) (**A**–**D**) or rats with bile-duct ligation (BDL) from day 14 of pregnancy (n = 6) (**E**–**H**). Values (means ± SEM) are expressed as individual bile acid species (**A**,**E**), total bile acids (**B**,**F**), taurine-conjugated bile acids (**C**,**G**) and unconjugated bile acids (**D**,**H**). *p < 0.05, **p < 0.01, comparing serum and milk by the paired *t* test.

Determination of the profile of different molecular species of BAs in lactating rats revealed that unconjugated BA forms (CA, DCA, CDCA, MCAs, and HDCA), but not tauroconjugated (TCA, TDCA, TCDCA, TMCAs and THDCA) nor glycoconjugated (GCA, GDCA and GCDCA) forms, were significantly more abundant in serum than in milk (Fig. 1A). Total BA concentrations were approximately 4-fold lower in milk than in serum (Fig. 1B). As the values for glycoconjugated BAs were negligible (Fig. 1A) and these of tauroconjugated BAs were similar in serum and milk (Fig. 1C), this difference was mainly accounted for by unconjugated BAs (Fig. 1D).

**Figure 2:**
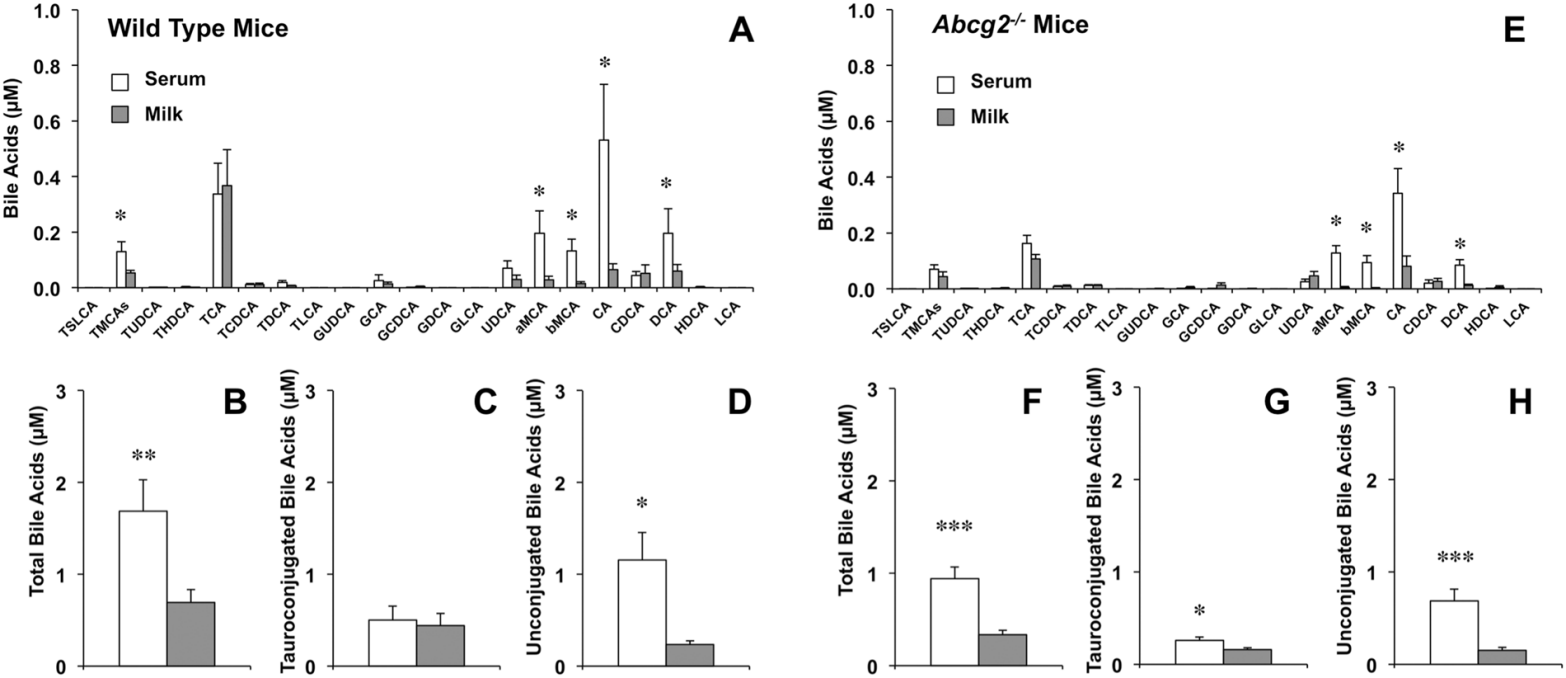
Bile acid concentrations as determined by HPLC-MS/MS in paired samples of serum and milk collected from lactating wild-type (n = 9) (**A–D**) or *Abcg2* ^−/−^ (n = 12) (**E**–**H**) mice on day 10 after delivery. Values (means ± SEM) are expressed as individual bile acid species (**A,E**), total bile acids (**B**,**F**), taurine-conjugated bile acids (**C**,**G**) and unconjugated bile acids (**D**,**H**). *p < 0.05, **p < 0.01, ***p < 0.001, comparing serum and milk by the paired *t* test.

These results support the existence of a selective barrier that hinders the passage of BAs from serum to milk, which seems to be more efficient for unconjugated forms of major BAs. In lactating mice, the pattern of BA species was also markedly different in serum and milk (Fig. 2A). Total BA concentrations were also higher in serum than in milk (Fig. 2B), which were also mainly due to differences in unconjugated forms (Fig. 2D) since concentrations of glycoconjugated BAs were negligible in both fluids (Fig. 2A) and these of tauroconjugated BAs were similar in serum and milk (Fig. 2C).

### Role of ABC proteins in BA handling by the mammary gland

To elucidate the molecular bases of the different profiles and levels of BAs between serum and milk in lactating rats and mice, the expression of ABC pumps able to transport BAs was analyzed by RT-QPCR in the mammary tissue of both species (Table 1).

**Table 1:** Relative mRNA levels of ABC transporters in the mammary gland of breastfeeding rats and mice.

Protein	Gene	mRNA Abundance (% of β-actin) × 10^3^			
		Rats		Mice	
		Control	BDL	Wild type	*Abcg2* ^−/−^
Bcrp	*Abcg2*	39846 ± 6891	45745 ± 6731	6979 ± 905	N.D.
Bsep	*Abcb11*	3.1 ± 0.9	5.2 ± 2.2	<1	<1
Mrp1	*Abcc1*	227 ± 32	297 ± 51	25 ± 9	19 ± 2
Mrp2	*Abcc2*	<1	<1	<1	<1
Mrp3	*Abcc3*	19 ± 4	29 ± 8	<1	<1
Mrp4	*Abcc4*	30 ± 4	63 ± 11^a^	16 ± 5	14 ± 1

Steady-state levels of mRNA in mammary gland tissue obtained from control and bile duct-ligated (BDL) rats, as well as wild-type and *Abcg2* ^−/−^ mice on day 10 ± 1 *post-partum*. Values are mean ± SEM, from measurements by RT-QPCR of samples from 6 animals per group, expressed as percentage of the abundance of mRNA of β-actin in each sample^a^. p < 0.05 as compared with control rats or wild-type mice by the Student *t*-test. N.D., not detected.

**Figure 3:**
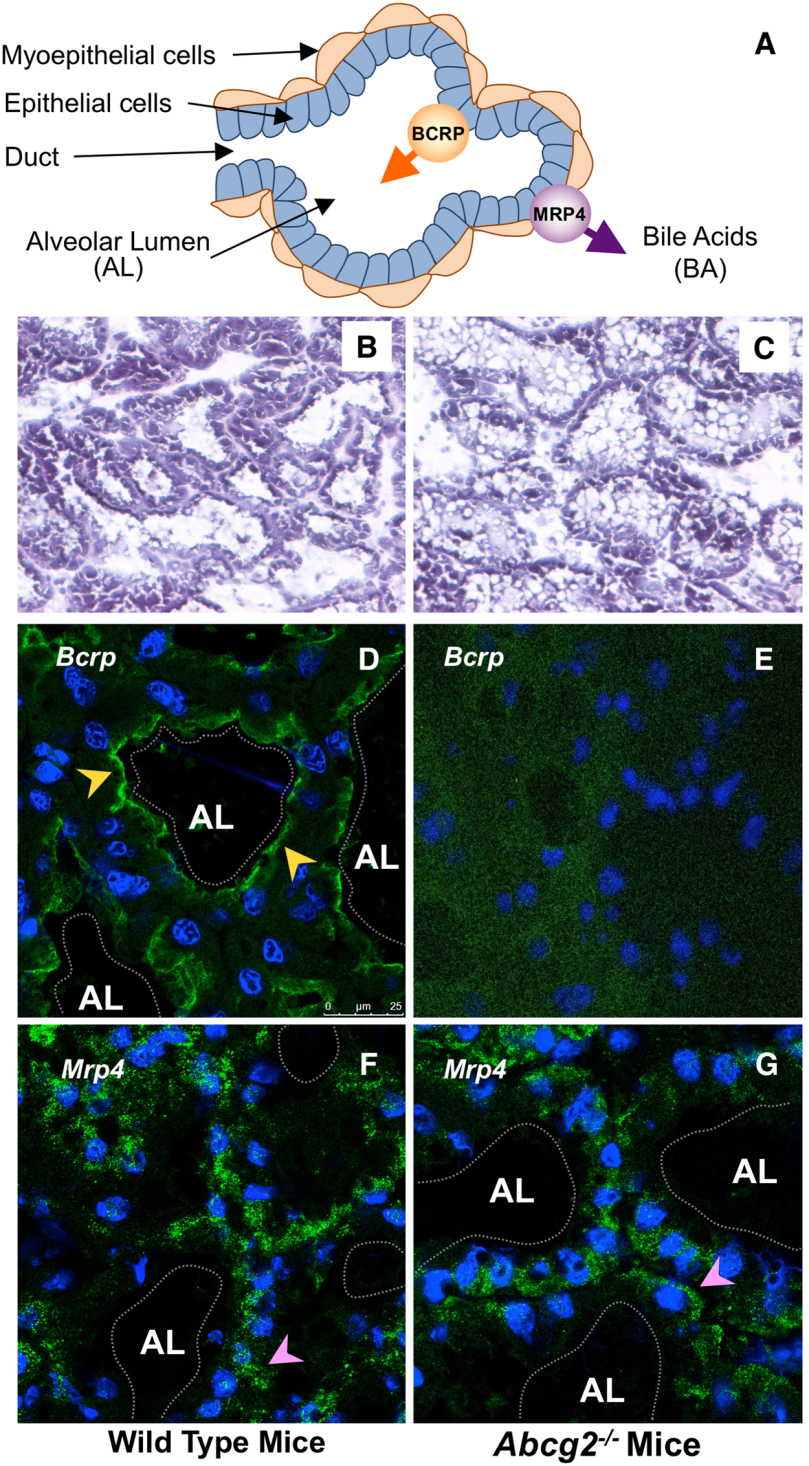
Schematic drawing of the mammary gland secretory unit (**A**). Representative images of histological sections of mammary gland tissue obtained from lactating mice on day 10 after delivery. Hematoxylin/eosin staining (original magnification: 20x) (**B**,**C**) and immunofluorescence analysis of Bcrp (**D**,**E**) and Mrp4 (**F**,**G**) were performed on mammary gland tissue obtained from lactating wild-type (**B**,**D**,**F**) and *Abcg2* ^−/−^ (**C**,**E**,**G**) mice. Arrowheads show the apical and basal localization of Bcrp (yellow) and Mrp4 (pink), respectively. AL: alveolar lumen.

Since the highest expression levels were these of Bcrp, to investigate its role in BA handling by the mammary gland, serum and milk of lactating *Abcg2* ^−/−^ mice were analysed 10 days *post-partum*. The absence of Bcrp in the mammary tissue of these animals was confirmed at the mRNA level (Table 1) and visualized by immunofluorescence (Fig. 3).

**Figure 4:**
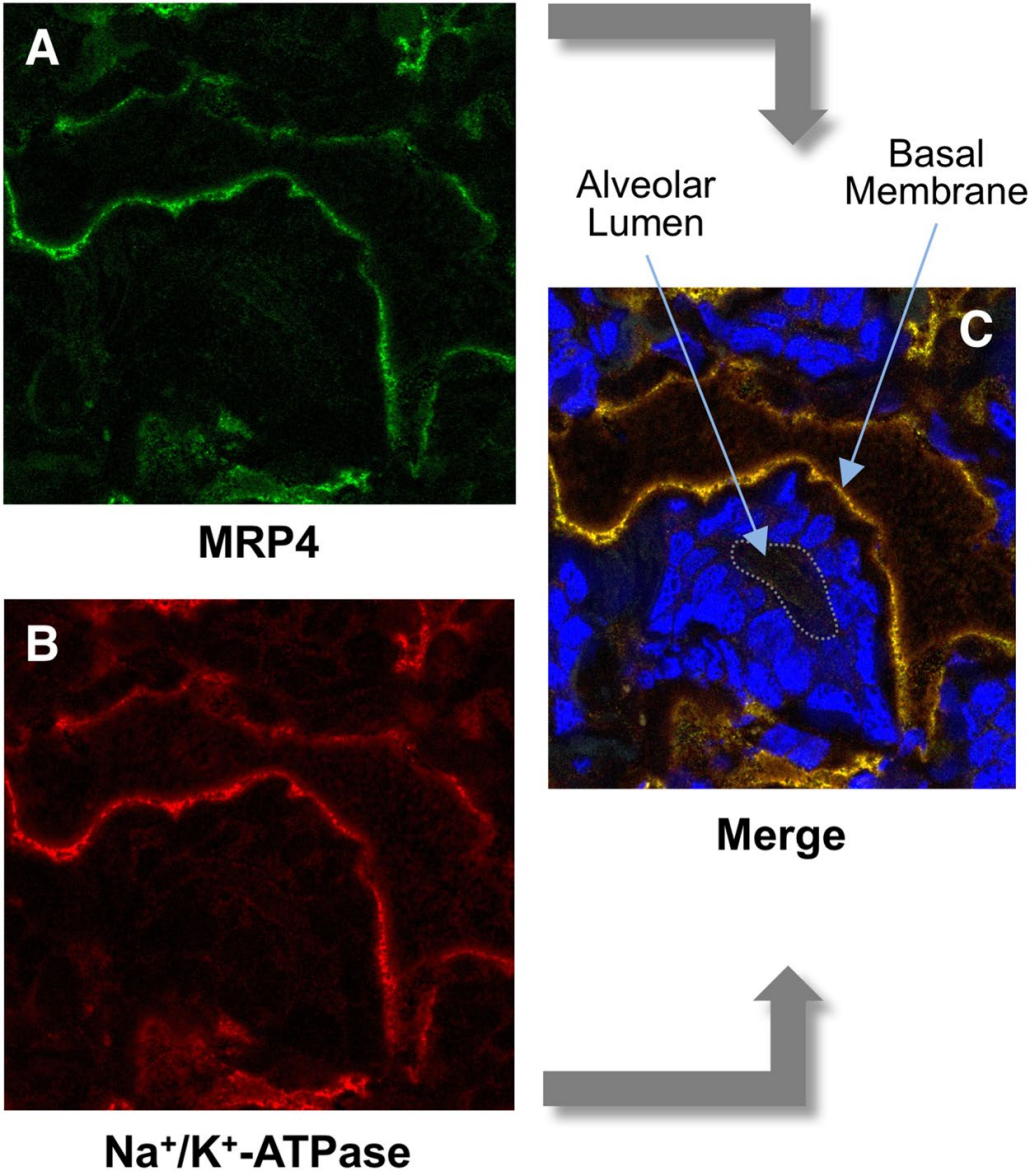
Immunofluorescence localization of MRP4 in breast tissue of a non-lactating woman. Cryosections were stained with anti-MRP4 (**A**, green) and anti-Na^+^/K^+^-ATPase (**B**, red) antibodies. Co-localization of both signals is shown in **C**. Nuclei were stained with DAPI.

The general structure of the secretory unit at the mammary gland is depicted in Fig. 3A. Figures 3B and C show representative images of histological sections of the mammary gland of wild-type and *Abcg2* ^−/−^ mice, respectively. As compared to the apical localization of Bcrp in wild-type animals (Fig. 3D), no signal was found in the mammary gland of *Abcg2* ^−/−^ mice (Fig. 3E). In contrast, Mrp4 was located at the basolateral membrane of both wild-type (Fig. 3F) and *Abcg2* ^−/−^ (Fig. 3G) mice. Since the anti-Mrp4 antibody worked better for human MRP4, and the presence of MRP4/Mrp4 in mammary gland was a matter of controversy, we evaluated the presence of this pump in healthy mammary gland tissue collected from a non-lactating woman. Clear co-localization of MRP4 with Na^+^/K^+^-ATPase at the basal membrane of this epithelium was detected (Fig. 4).

**Figure 5:**
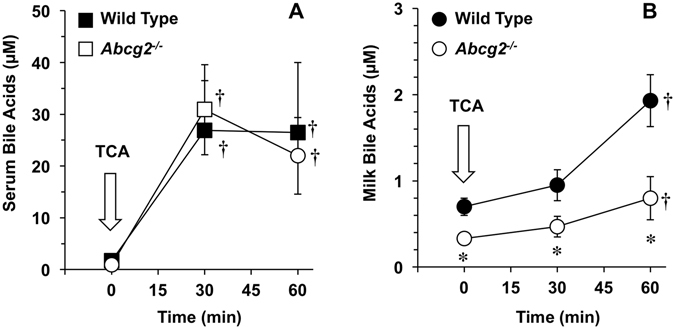
Bile acid concentrations as determined by HPLC-MS/MS in paired samples of serum (**A**) and milk (**B**) collected from lactating wild-type and *Abcg2* ^−/−^ mice on day 10 after delivery. At min 0 the animals received (i.p.) 5 µmol taurocholic acid (TCA). Owing to the impossibility of repeated sampling from the same mice, blood and milk samples were collected at min 0, 30 or 60 after TCA administration in different sets of animals (n = 5 per group). Values are means ± SEM. *p < 0.05, comparing wild-type and *Abcg2* ^−/−^ mice. ^†^p < 0.05, compared with basal values before TCA administration. Bonferroni method of multiple-range testing was used for comparisons.

BA concentrations in serum and milk were lower in *Abcg2* ^−/−^ than in wild-type mice (Figs 2B and F). As in wild-type mice (Fig. 2A), in lactating *Abcg2* ^−/−^ mice, the pattern of BA species in serum and milk was also different (Fig. 2E). In this case, the higher values of total BA concentrations in serum (Fig. 2F) were due to differences in both tauroconjugated (Fig. 2G) and unconjugated (Fig. 2H) BA species. In order to elucidate whether the lower BA levels in milk of *Abcg2* ^−/−^ mice were merely a reflection of the lower serum concentrations found in these animals, and to evaluate the importance of the absence of Bcrp in the putative barrier for BAs present in breast tissue, a transient hypercholanemia was induced by TCA administration (5 µmol, i.p.). This resulted in a marked increase (>15-fold) in serum BAs that was similar in wild-type and *Abcg2* ^−/−^ mice (Fig. 5A). TCA-induced maternal hypercholanemia was accompanied by a moderate (<3-fold) rise in milk BA levels that was similar in both groups of animals (Fig. 5B). These results suggest that: i) the basal secretion of BAs into milk is reduced in absence of Bcrp expression; ii) there is a barrier that limits the passage of BAs from blood to milk; and iii) such barrier is maintained even in the absence of Bcrp expression.

**Figure 6:**
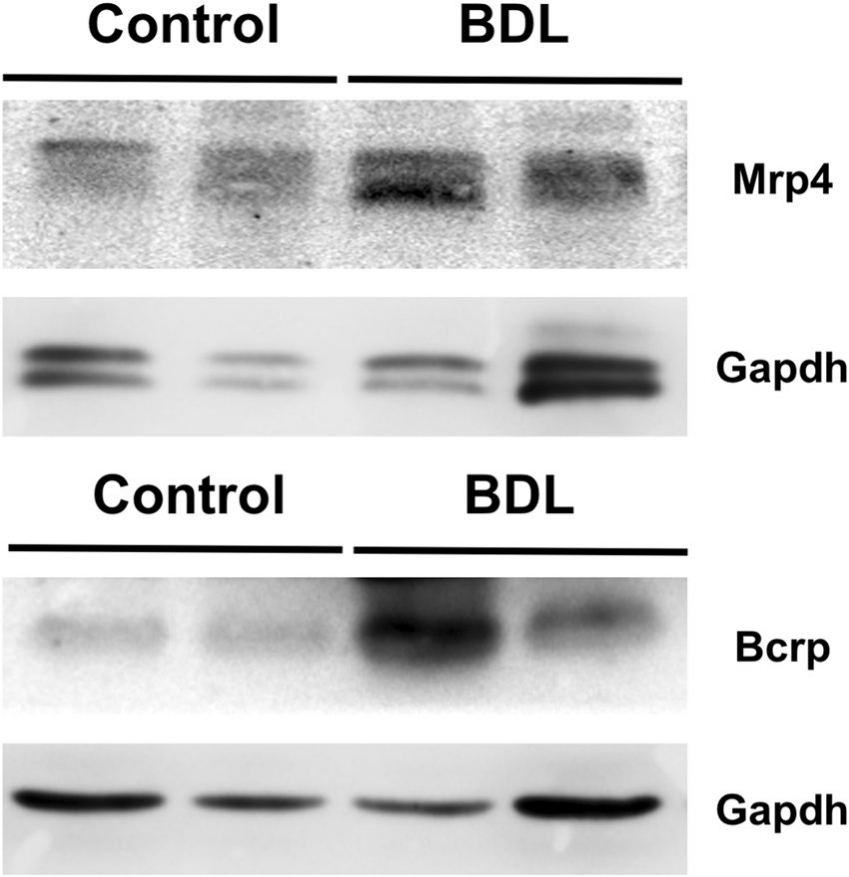
Representative western blot analysis of Mrp4 and Bcrp in mammary gland lysates obtained from two control and two bile duct-ligated (BDL) rats on day 10 ± 1 *post-partum*. Detection of GAPDH served as a loading control.

The analysis of other ABC pumps able to transport BAs in rat and mouse mammary gland revealed that the expression of apical pumps responsible for the secretion of BAs by hepatocytes into bile, such as Bsep and Mrp2, was negligible in mammary gland, whereas basolateral pumps, such as Mrp1 and Mrp4, involved in the efflux of BAs from hepatocyte to blood under cholestatic conditions^27, 28^, were clearly expressed in the mammary tissue of both species. Mrp3 mRNA was also detected in rats but at lower levels (Table 1). Among these ABC pumps, only Mrp4 responded to cholestasis in a similar way to that reported in hepatocytes. Hence, Mrp4 was significantly up-regulated when maternal cholestasis was imposed on rats by BDL from day 14 of gestation. Results from western blot analyses were consistent with the observed trend toward enhanced abundance of both Bcrp and Mrp4 in the mammary tissue of BDL rats (Fig. 6). The location of MRP4/Mrp4 at the basolateral membrane of mammary alveolar epithelial cells in mice (Fig. 3F–G) and in human tissue (Fig. 4) supports a functional role of MRP4/Mrp4 in the blood-milk barrier for BAs.

### Evaluation of the blood-milk BA barrier

To investigate the effect of maternal hypercholanemia on the balance of BAs across the mammary epithelium, BA concentrations in milk were determined in pregnant rats with obstructive cholestasis from day 14 of gestation. At day 10 *post-partum*, the most abundant molecular species in both serum and milk were primary BAs (TMCAs > TCA > TCDCA) (Fig. 1E). Total BA concentrations in serum (Fig. 1F) were markedly increased (22-fold) compared with the concentrations found in control non-cholestatic mothers (Fig. 1B). Hypercholanemia was accompanied by a 15-fold increase in the concentrations of BAs in milk. The milk-to-serum ratio of BAs was reduced from 0.29 ± 0.09 (in control group) to 0.18 ± 0.02 (in BDL animals). As could be expected in animals with complete biliary obstruction in which BA deconjugation by intestinal bacteria has been prevented, in BDL mothers, tauroconjugated BAs predominated in both serum and milk (Fig. 1G), whereas unconjugated BAs accounted for less than 1% of total serum BAs (Fig. 1H).

### Effect of breastfeeding on neonatal BA homeostasis

**Figure 7:**
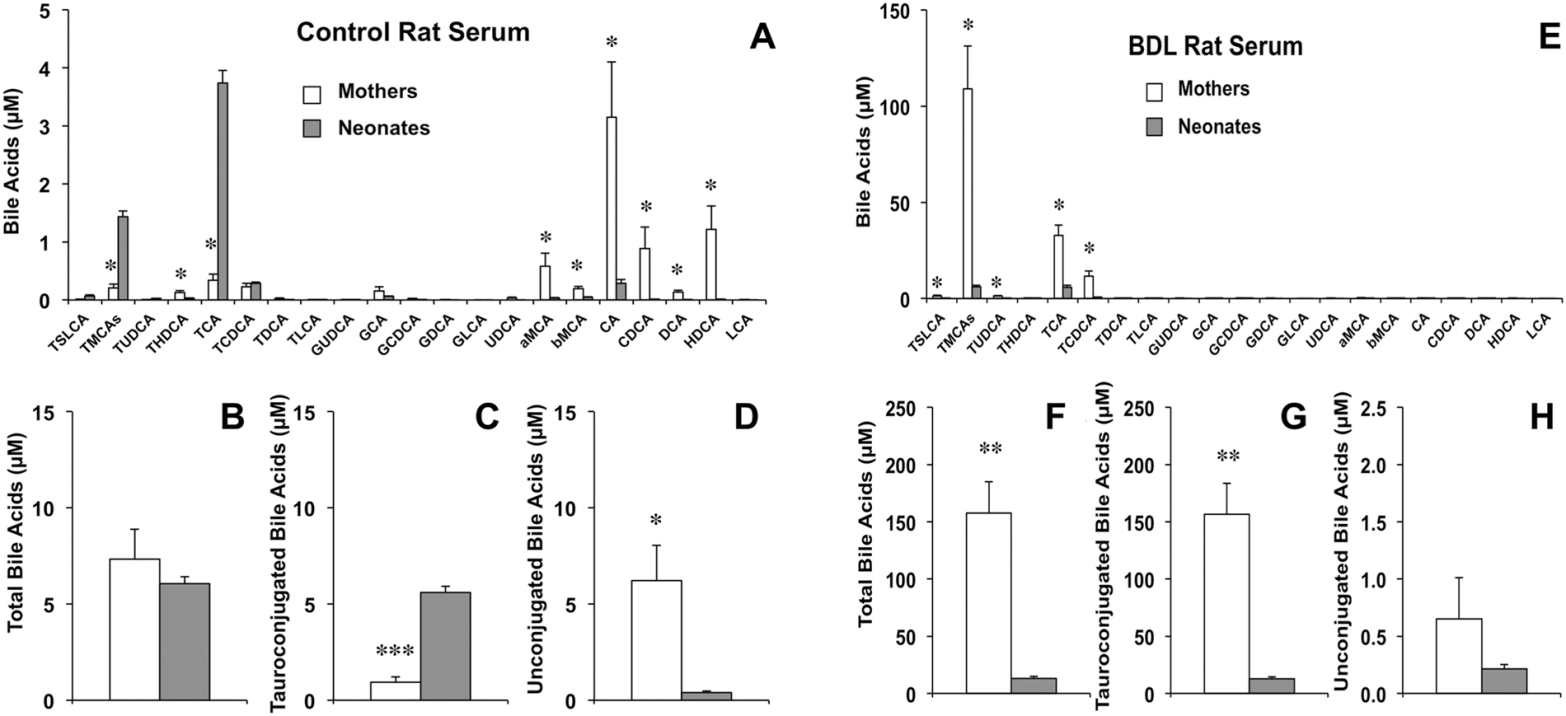
Comparison of serum bile acid concentrations between lactating rats and their offspring. Bile acid concentrations were determined by HPLC-MS/MS in serum samples collected on day 10 after delivery from healthy lactating rats (n = 6) and their pups (n = 14) (**A**–**D**) or from rats with bile-duct ligation (BDL) from day 14 of pregnancy (n = 6) and their pups (n = 14) (**E**–**H**). Values (means ± SEM) are expressed as individual bile acid species (**A,E**), total bile acids (**B,F**), taurine-conjugated bile acids (**C**,**G**) and unconjugated bile acids (**D**,**H**). *p < 0.05, **p < 0.01, ***p < 0.001, comparing maternal with neonatal serum by the paired *t* test.

In control animals, when serum BA profiles in the neonates and their mothers were compared (Fig. 7A), no significant differences regarding total BAs were found (Fig. 7B). However, tauroconjugated BAs were more abundant in neonatal serum (Fig. 7C), whereas the opposite occurred for unconjugated forms, which predominated in maternal serum (Fig. 7D). In contrast, in the BDL group serum BA profiles were markedly different (Fig. 7E). Total serum BA concentrations were dramatically higher in mothers than in their neonates (Fig. 7F), which reflected the differences in the levels of tauroconjugated species (Fig. 7G), since unconjugated forms were >100-fold lower (Fig. 7H).

**Figure 8:**
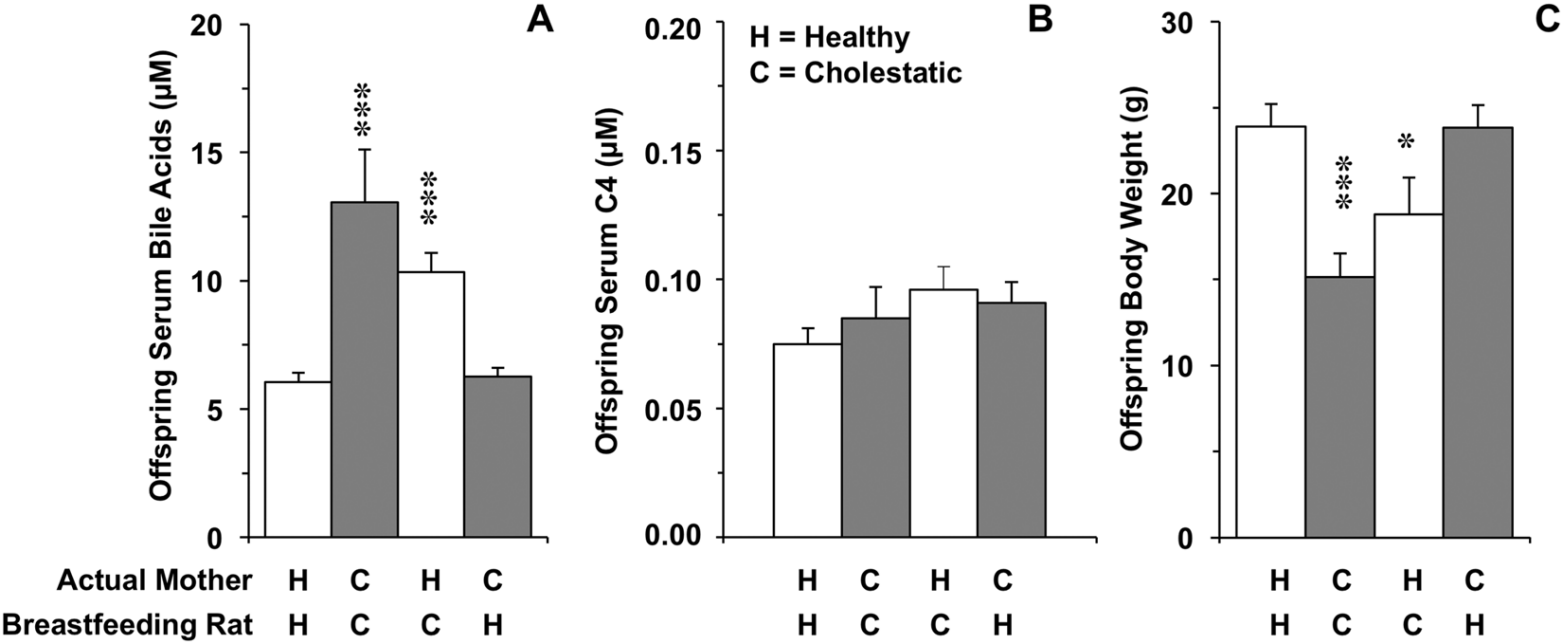
Serum concentrations of total bile acids (**A**) and bile acid precursor 7α-hydroxy-4-cholesten-3-one or C4 (**B**) and body weight (**C**) of the offspring of healthy control (**H**) and cholestatic (**C**) lactating rats on day 10 after delivery. Obstructive cholestasis was imposed on the mothers by bile-duct ligation on day 14 of pregnancy. In two additional groups litters and mothers were exchanged immediately after delivery in order to impose breastfeeding of pups born from H rats by C rats and pups born from C rats by H rats. Bile acids and C4 were measured by HPLC-MS/MS. Values are means ± SEM of serum samples obtained from 8 to 14 pups belonging to 5 to 7 different litters in each case, *p < 0.05, ***p < 0.001, compared with control by the Bonferroni method of multiple-range testing.

In previous studies we reported that, at delivery, newborns of BDL rats were smaller and had higher BA serum levels than those born from control mothers^10, 29^. These differences still persisted at day 10 of lactation, when serum BA concentrations were 2-fold in BDL pups than in control pups of the same age (Fig. 8A), whereas body weight was lower (Fig. 8C). In the rat model of obstructive cholestasis used in this study, the fetuses were exposed to maternal hypercholanemia during the last week of pregnancy and then fed with BA-enriched milk for 10 days. To ascertain whether elevated serum BA levels found in these animals were due, in part, to an additional supply of these compounds with the maternal milk, the litters and mothers were immediately exchanged after birth. The neonates born from healthy mothers (controls) and subsequently fed by BDL rats showed higher serum BA levels than those fed by the control animals (Fig. 8A). Conversely, the offspring of mothers with cholestasis that were fed by control rats for 10 days showed serum BA levels similar to those of the control group (Fig. 8A). No significant differences were found in the hepatic expression of several BA-related genes, such as key biosynthetic enzymes (Cyp7a1 and Cyp27a1) and nuclear receptors (Fxr and Shp), among neonates of the four groups (Supplementary Table 3). Moreover, the concentrations of 7α-hydroxy-4-cholesten-3-one (C4) in neonatal serum, which was determined as an indirect measurement of liver Cyp7a1 activity, were similar in all groups (Fig. 8B), suggesting that elevation of serum BA levels in BDL neonates was not due to enhanced synthesis by the neonatal liver. Interestingly, body weight was markedly dependent on whether breastfeeding mothers were healthy controls or cholestatic rats, regardless of if the pups were born from healthy or BDL rats (Fig. 8C).

## Discussion

The results of the present study support the hypothesis that ABC proteins are involved in the secretory/barrier function for BAs across the mammary epithelium in an asymmetric manner, i.e., BCRP/Bcrp may play a moderate role in BA secretion into milk, whereas MRP4/Mrp4 is involved in a strong barrier limiting BA traffic from blood to milk even in the presence of maternal hypercholanemia.

The high expression of Bcrp in the mammary gland of lactating rats and mice (Table 1), as well as in cows and humans^20^, suggests that it could play a major role in BA secretion into milk. The expression of this pump in mammary epithelial tissue of non-pregnant female mice is negligible, is highly induced during late pregnancy and even more during breastfeeding, and rapidly declines when lactation stops^20^. Using Bcrp knockout mice, a role for this ABC protein in the excretion of several drugs, such as topotecan, cimetidine, among others^20^, and vitamins (riboflavin, biotin)^23^, into milk has been described. However, its contribution to the secretion of known substrates of this pump, such as folate, dehydroepiandrosterone sulfate and vitamin B_12_ has been ruled out^19, 20^. Regarding BAs, which are also transported by this pump^24^, we found a moderate dependence of BA secretion on the expression of Bcrp in mouse mammary gland. The location of the protein at the apical membrane of alveolar cells and the observation that concentrations of BAs in milk from *Abcg2* ^−/−^ mice were significantly lower than in wild-type animals are consistent with a secretory role for this pump. Since our findings confirmed the moderate (30–50%) reduction in serum BA levels in *Abcg2* ^−/−^ mice that had been previously reported^24, 30^, one could speculate that the lower levels of BAs in the milk of Abcg2^−/−^ mice merely reflect the decrease in serum of BA concentrations. However, the present study demonstrates that both in wild-type and in *Abcg2* ^−/−^ mice BA secretion into milk was markedly insensitive to changes in maternal serum BA concentrations.

To mimic the situation of hypercholanemia that occurs during late gestation in ICP patients, our group, as well as others, have repeatedly used the experimental model of complete obstructive cholestasis during rat gestation^10, 11, 29^. In the present study, we analysed wild-type and BDL animals at 10 ± 1 *post-partum*, since this is approximately the halfway point of the lactation period; weaning usually takes place at day 21 in rats. The BA levels found in the milk of healthy lactating rats were in the micromolar range, being similar to those reported in human colostrum^15^ but lower than in serum. In ICP patients, it has been shown that BA concentrations in milk samples collected two days after delivery were still 30-fold higher than those of healthy women, although still lower (50%) than those measured in their serum^15^. In BDL lactating rats, hypercholanemia was also accompanied by an increase in milk BA levels, but not as marked as that observed in serum. Also, when a transient hypercholanemia was induced by TCA administration in lactating mice, the milk-to-serum ratio of BAs was dramatically reduced from 50–60% to <10%, as the rapid rise in serum was accompanied by an only mild increase in milk. These findings, together with the marked difference in the profiles of BA molecular species found in serum and milk of control rats and mice, support the existence of a selective barrier that hinders the free passage of BAs from blood to milk. It must be noted that, although BDL experiments in knockout mice would be of interest to confirm this hypothesis, these could not be performed because of the high rate of abortions induced by BDL in mice (data not shown).

Among the basolateral ABC pumps able to transport BAs, several members of the ABC family could be considered. However, only Mrp1 and Mrp4 were expressed in the mammary tissue of both rats and mice. Additionally, it is important to consider that lactation is a dynamic process and stage-dependent changes in transporter expression have been reported. Thus, Mrp1 expression in rat mammary gland decreased throughout the lactation period, and was reduced at day 10 by 60% as compared to levels found at term^31^. Furthermore, in both lactating and non-lactating epithelial cells isolated from human mammary glands, mRNA of MRP1, but not of MRP3 or MRP4 could be detected^32^. The authors of that study suggested that the typical basolateral localization of MRP1 at the plasma membrane pointed to a protective role of this pump in the lactating mammary gland by extruding potentially toxic agents from this tissue, thus reducing their levels in milk^32^. Our results support a similar role for MRP4 regarding BAs, because when the mRNA, obtained directly from rat and mouse mammary tissue, was measured by RT-QPCR or immunofluorescence analysis was carried out in mammary gland of lactating mice and non-lactating women we could clearly detect MRP4 expression, suggesting that cell isolation and purification procedures may have hampered the detection of this protein in previous studies^32^. MRP4 is thought to play an important compensatory role in cholestasis by exporting BAs from hepatocytes to blood across the basolateral plasma membrane, enabling the excretion of these compounds by the kidneys. Accordingly, in BDL rats, Mrp4 is up-regulated in liver but down-regulated in kidney^28^. Up-regulation of MRP4, but not MRP3, has also been described in cholestatic liver disease in humans^33^.

The biological function of BAs in milk is not known. The bile salt-dependent lipase (BSL) present in human milk has been related to the higher coefficients of fat absorption observed in breast-fed *vs*. formula-fed newborns^34^. Milk BSL also plays a defensive role in the digestive tract of infants by contributing to the protection of the baby from parasites such as *Giardia lamblia* ^35^. However, the BA concentrations found in milk are too low to significantly enhance the enzymatic activity of BSL^36^. As for the contribution of milk BAs to the total BA pool in newborns, higher concentrations of these compounds were detected in the serum of pups fed by cholestatic rats, even if they were born from control healthy mothers. Conversely, in the offspring of cholestatic rats, serum BA levels were normalized after breastfeeding for 10 days by healthy lactating rats. Thus, it appears that BAs supplied with the milk are quantitatively important because they are effectively taken up and incorporated into the BA pool present in the suckling pups. Therefore, pathological high levels of BAs in maternal milk could result in an enhanced BA pool size in newborns. The actual repercussion on neonatal physiology is unknown, but it should be considered that at this ontogenic stage the enterohepatic circulation is still immature.

In conclusion, our results support the existence of a balance between BA secretion into milk across the apical membrane of mammary gland epithelial cells, in which Bcrp seems to be involved, and the efflux in the opposite direction across the basal membrane of these cells that is likely to involve Mrp4, which constitutes an efficient blood-milk barrier for BAs. This latter mechanism seems to play a protective role for the offspring by limiting milk BA concentrations in situations of maternal cholestasis.

## Methods

### Chemicals

BAs used as standards in HPLC-MS/MS analyses: cholic acid (CA), chenodeoxycholic acid (CDCA), deoxycholic acid (DCA), lithocholic acid (LCA), ursodeoxycholic acid (UDCA), α-muricholic acid (αMCA), β-muricholic acid (βMCA) and hyodeoxycholic (HDCA) acid, as well as their taurine-conjugated (TCA, TCDCA, TDCA, TLCA, TUDCA, TαMCA, TβMCA, THDCA) and glycine-conjugated (GCA, GCDCA, GDCA, GLCA, GUDCA) forms, taurosulfolithocholic acid (TSLCA) and 7α-hydroxy-4-cholesten-3-one (C4) were from Sigma-Aldrich (Madrid, Spain). According to the supplier the purity of these compounds was ≥97%. All other chemicals were of analytical grade.

### Animals

Pregnant Wistar rats from the University of Salamanca Animal House Facility (Salamanca, Spain) and pregnant FVB wild-type mice and homozygous constitutive Bcrp knockout (*Abcg2* ^−/−^) mice (FVB.129 P^-Abcg2tm1Ahs^) from Taconic Farms (Germantown, NY) were used. In all cases, litters were adjusted to 8 neonates immediately after delivery. The animals received humane care as outlined in the National Institutes of Health guidelines for the care and use of laboratory animals (Guide for the Care and Use of Laboratory Animals, 8^th^ edition, 2011). The experimental protocols were approved by the Ethical Committee for the Use of Laboratory Animals of the University of Salamanca.

### Experiments in lactating rats

To investigate the effect of maternal cholestasis on BA levels in milk, as well as on the expression of ABC proteins in the lactating breast tissue, complete obstruction of the maternal common bile duct was imposed and maintained in rats from day 14 of gestation, as described elsewhere^10^. Sham operated pregnant/lactating animals were used as controls. On day 10 ± 1 *post-partum* mothers were anaesthetised with sodium pentobarbital (50 mg/kg b.w., i.p.) to collect milk (by gentle aspiration of the mammary glands) and blood (from the inferior cava vein). Oxytocin (0.2 IU, i.p.) was administered 10 min before sampling to stimulate milk ejection^37^. Before euthanizing the animals by anaesthesia overdosing, tissue samples from mammary glands were collected and stored in RNA*later* reagent until analysis. On the same day, blood samples were obtained from the offspring by decapitation under anaesthesia with pentobarbital. Blood was allowed to clot by 30 min at room temperature before obtaining serum by centrifugation.

### Experiments in lactating mice

To evaluate the role of Bcrp in BA transport into milk, wild-type and *Abcg2* ^−/−^ lactating mice were used. On day 10 ± 1 *post-partum* mothers were anaesthetised with sodium pentobarbital (50 mg/kg b.w., i.p.) to collect milk (by gentle aspiration of the mammary glands) and blood (from the inferior cava vein). Oxytocin was i.p. administered to stimulate milk ejection. Based on previous experience in our laboratory (data not shown) in some animals, taurocholic acid (TCA, 5 µmol) was injected (i.p.) to induce a transient hypercholanemia, which was later confirmed. In different sets of animals, to avoid repeated sampling from the same mice, blood and milk samples were then collected 30 or 60 min after TCA administration. At the end of the experiments, mammary gland samples were collected and stored in RNA*later* reagent or frozen in liquid nitrogen and stored at −80 °C until measurement of gene/protein expression.

### Analyses of BAs in serum and milk

Silica-based bonded phase cartridges (Sep-Pack Plus C18, Waters, Madrid) were used to extract BAs from serum and milk samples. Validation assays were carried out to confirm the absence of interfering matrix effects. Methanolic extracts were analysed using an adaptation^38^ of a previously described method for BA measurement by HPLC-MS/MS^39^ on a 6420 Triple Quad LC/MS device (Agilent Technologies, Santa Clara, CA). The BA precursor C4 was determined in serum after acetonitrile precipitation/extraction^40^ by a modification of an HPLC-MS/MS method^41^.

### RT-QPCR

Total RNA extraction was carried out using the illustra RNAspin Mini RNA Isolation Kit (GE Healthcare Life Sciences, Barcelona, Spain) and retrotranscription (RT) using a high-capacity cDNA reverse transcription kit (Applied Biosystems, Madrid). Real-time quantitative PCR (QPCR) was performed using AmpliTaq Gold polymerase (Applied Biosystems) in an ABI Prism 7300 Sequence Detection System (Applied Biosystems) with the following thermal conditions: a single cycle at 50 °C for 2 min and at 95 °C for 10 min followed by 40 cycles at 95 °C for 15 s and at 60 °C for 60 s. The oligonucleotide sequences of the primers used to carry out QPCR are shown in Supplementary Tables 1 and 2. The results of mRNA abundance of the target genes in each sample were normalized on the basis of β-actin mRNA abundance. Detection of amplification products was carried out using SYBR Green I. Total RNA from control male mouse or rat liver was used as a calibrator, as appropriate. Results were expressed as the percentage of the abundance of mRNA of β-actin in each sample.

### Western blot

Immunoblotting analyses of mammary gland lysates were carried out in 7.5% SDS-PAGE, loading 50 µg protein per lane with β-mercaptoethanol or 100 μg of protein per lane without β-mercaptoethanol for Bcrp and Mrp4, respectively. Blots were probed with the following antibodies diluted in TBS-Tween: primary monoclonal antibodies against Bcrp (BXP-21, 1:500, Abcam) and GAPDH (6C5, 1:1000, Santa Cruz Biotechnology) and goat polyclonal antibody against Mrp4 (NB-100-1471, 1:200, Novus Biologicals). Immunoreactive protein bands were visualized by an enhanced chemiluminescence (ECL) detection system (Amersham, GE Healthcare, Barcelona) after incubation with appropriate secondary antibodies (IgG-HRP-linked) (1:2000, Santa Cruz Biotechnology).

### Immunofluorescence assays

Immunostaining was carried out on 5-µm cryosections of mice mammary gland. Human non-lactating breast tissue obtained from routine biopsies with confirmed absence of malignancy, from the Biobank of the University Hospital of Salamanca, was used to confirm the membrane expression of MRP4. The study was approved by the Medical Ethics Committee of the University Hospital and conducted according to the principles expressed in the Declaration of Helsinki. Slides were air-dried before fixation and permeabilization with ice-cold methanol. The following primary antibodies were used diluted in 2% foetal calf serum in PBS: rat monoclonal against mouse Bcrp (BXP-9, Enzo Life Sciences, Lausanne, Switzerland); goat anti-human/rat MRP4/Mrp4 (NB-100-1471, Novus Biologicals), and rabbit anti-Na^+^/K^+^-ATPase (H-300, Santa Cruz Biotechnology). After washing, the slides were incubated with the appropriate Alexa 488- or Alexa 594- (1:1000) conjugated antibodies (Life Technologies), and nuclei were counterstained with DAPI (10 µM). Fluorescence staining was visualized using a Leica TCS SP2 confocal microscope.

### Histological studies

Mice mammary gland cryosections were immersed in 4% paraformaldehyde and later embedded in paraffin. The slices (5 µm) were stained with hematoxylin & eosin^42^.

### Statistical analysis

Data are presented as means ± SEM. After performing an ANOVA test, the Student *t*-test, the paired-*t* test or the Bonferroni method of multiple-range testing were used, as appropriate, to calculate the statistical significance of differences among groups.

## Electronic supplementary material

Supplementary Tables 1, 2 and 3

## References

[1] Westbrook RH, Dusheiko G, Williamson C (2016). Pregnancy and liver disease. J Hepatol.

[2] Glantz A, Marschall HU, Mattsson LA (2004). Intrahepatic cholestasis of pregnancy: Relationships between bile acid levels and fetal complication rates. Hepatology.

[3] Geenes V (2014). Association of severe intrahepatic cholestasis of pregnancy with adverse pregnancy outcomes: a prospective population-based case-control study. Hepatology.

[4] Brouwers L (2015). Intrahepatic cholestasis of pregnancy: maternal and fetal outcomes associated with elevated bile acid levels. Am J Obstet Gynecol.

[5] Estiu MC (2017). Relationship between early onset severe intrahepatic cholestasis of pregnancy and higher risk of meconium-stained fluid. PLoS One.

[6] Dixon PH, Williamson C (2016). The pathophysiology of intrahepatic cholestasis of pregnancy. Clin Res Hepatol Gastroenterol.

[7] Arrese M, Macias RI, Briz O, Perez MJ, Marin JJ (2008). Molecular pathogenesis of intrahepatic cholestasis of pregnancy. Expert Rev Mol Med.

[8] EASL (2009). Clinical Practice Guidelines: management of cholestatic liver diseases. J Hepatol.

[9] Estiu MC (2015). Effect of ursodeoxycholic acid treatment on the altered progesterone and bile acid homeostasis in the mother-placenta-foetus trio during cholestasis of pregnancy. Br J Clin Pharmacol.

[10] Monte MJ (1996). Reversible impairment of neonatal hepatobiliary function by maternal cholestasis. Hepatology.

[11] Macias RI (2005). Long-term effect of treating pregnant rats with ursodeoxycholic acid on the congenital impairment of bile secretion induced in the pups by maternal cholestasis. J Pharmacol Exp Ther.

[12] Papacleovoulou G (2013). Maternal cholestasis during pregnancy programs metabolic disease in offspring. J Clin Invest.

[13] Forsyth JS, Ross PE, Bouchier IA (1983). Bile salts in breast milk. Eur J Pediatr.

[14] Vítek L, Zelenková M, Brůha R (2010). Safe use of ursodeoxycholic acid in a breast-feeding patient with primary biliary cirrhosis. Dig. Liver Dis..

[15] Brites D, Rodrigues CM (1998). Elevated levels of bile acids in colostrum of patients with cholestasis of pregnancy are decreased following ursodeoxycholic acid therapy [see comemnts]. J Hepatol.

[16] Pusl T, Beuers U (2007). Intrahepatic cholestasis of pregnancy. Orphanet J Rare Dis.

[17] Murphy K (2017). The Composition of Human Milk and Infant Faecal Microbiota Over the First Three Months of Life: A Pilot Study. Sci Rep.

[18] Mennone A (2006). Mrp4^−/−^ mice have an impaired cytoprotective response in obstructive cholestasis. Hepatology.

[19] van Herwaarden AE, Schinkel AH (2006). The function of breast cancer resistance protein in epithelial barriers, stem cells and milk secretion of drugs and xenotoxins. Trends Pharmacol Sci.

[20] Jonker JW (2005). The breast cancer resistance protein BCRP (ABCG2) concentrates drugs and carcinogenic xenotoxins into milk. Nat Med.

[21] Merino G, Jonker JW, Wagenaar E, van Herwaarden AE, Schinkel AH (2005). The breast cancer resistance protein (BCRP/ABCG2) affects pharmacokinetics, hepatobiliary excretion, and milk secretion of the antibiotic nitrofurantoin. Mol Pharmacol.

[22] Mao Q, Unadkat JD (2015). Role of the breast cancer resistance protein (BCRP/ABCG2) in drug transport–an update. AAPS J.

[23] Vlaming ML, Lagas JS, Schinkel AH (2009). Physiological and pharmacological roles of ABCG2 (BCRP): recent findings in Abcg2 knockout mice. Adv Drug Deliv Rev.

[24] Blazquez AG (2012). Characterization of the role of ABCG2 as a bile acid transporter in liver and placenta. Mol Pharmacol.

[25] Allikmets R, Schriml LM, Hutchinson A, Romano-Spica V, Dean M (1998). A human placenta-specific ATP-binding cassette gene (ABCP) on chromosome 4q22 that is involved in multidrug resistance. Cancer Res.

[26] Serrano MA (2007). Expression in human trophoblast and choriocarcinoma cell lines, BeWo, Jeg-3 and JAr of genes involved in the hepatobiliary-like excretory function of the placenta. Placenta.

[27] Trauner M, Boyer JL (2003). Bile salt transporters: molecular characterization, function, and regulation. Physiol Rev.

[28] Denk, G. U. *et al*. Multidrug resistance-associated protein 4 is up-regulated in liver but down-regulated in kidney in obstructive cholestasis in the rat. *J Hepatol* **40**, 585–591, doi:S0168827803006354 [pii] (2004). 10.1016/j.jhep.2003.12.001 15030973

[29] Macias RI, Jimenez S, Serrano MA, Monte MJ, Marin JJ (2006). Effect of maternal cholestasis and treatment with ursodeoxycholic acid on the expression of genes involved in the secretion of biliary lipids by the neonatal rat liver. Life Sci.

[30] Mennone A, Soroka CJ, Harry KM, Boyer JL (2010). Role of breast cancer resistance protein in the adaptive response to cholestasis. Drug Metab Dispos.

[31] Gilchrist SE, Alcorn J (2010). Lactation stage-dependent expression of transporters in rat whole mammary gland and primary mammary epithelial organoids. Fundam Clin Pharmacol.

[32] Alcorn J, Lu X, Moscow JA, McNamara PJ (2002). Transporter gene expression in lactating and nonlactating human mammary epithelial cells using real-time reverse transcription-polymerase chain reaction. J Pharmacol Exp Ther.

[33] Keitel V (2005). Expression and localization of hepatobiliary transport proteins in progressive familial intrahepatic cholestasis. Hepatology.

[34] Lindquist S, Hernell O (2010). Lipid digestion and absorption in early life: an update. Curr Opin Clin Nutr Metab Care.

[35] Gillin FD (1987). Giardia lamblia: the role of conjugated and unconjugated bile salts in killing by human milk. Exp Parasitol.

[36] Blackberg L, Hernell O (1993). Bile salt-stimulated lipase in human milk. Evidence that bile salt induces lipid binding and activation via binding to different sites. FEBS Lett.

[37] Merino G (2010). *In vivo* inhibition of BCRP/ABCG2 mediated transport of nitrofurantoin by the isoflavones genistein and daidzein: a comparative study in Bcrp1 (−/−) mice. Pharm Res.

[38] Nytofte NS (2011). A homozygous nonsense mutation (c.214C− > A) in the biliverdin reductase alpha gene (BLVRA) results in accumulation of biliverdin during episodes of cholestasis. J Med Genet.

[39] Ye L, Liu S, Wang M, Shao Y, Ding M (2007). High-performance liquid chromatography-tandem mass spectrometry for the analysis of bile acid profiles in serum of women with intrahepatic cholestasis of pregnancy. J Chromatogr B Analyt Technol Biomed Life Sci.

[40] Lenicek M, Vecka M, Zizalova K, Vitek L (2016). Comparison of simple extraction procedures in liquid chromatography-mass spectrometry based determination of serum 7alpha-hydroxy-4-cholesten-3-one, a surrogate marker of bile acid synthesis. J Chromatogr B Analyt Technol Biomed Life Sci.

[41] Steiner C, von Eckardstein A, Rentsch KM (2010). Quantification of the 15 major human bile acids and their precursor 7alpha-hydroxy-4-cholesten-3-one in serum by liquid chromatography-tandem mass spectrometry. J Chromatogr B Analyt Technol Biomed Life Sci.

[42] Herraez E (2014). Role of macrophages in bile acid-induced inflammatory response of fetal lung during maternal cholestasis. J Mol Med (Berl).

