# Supplementary material for: Lactation during cholestasis: Role of ABC proteins in bile acid traffic across the mammary gland
Source: Sci Rep. 2017 Aug 7;7:7475. doi: 10.1038/s41598-017-06315-8 (PMC5547141; doi:10.1038/s41598-017-06315-8)
Supplement: Supplementary file 1 — Supplementary Tables 1, 2 and 3 [file 41598_2017_6315_MOESM1_ESM.pdf]

SUPPLEMENTARY INFORMATION

Lactation during cholestasis: Role of ABC proteins in bile acid traffic across the mammary gland

Alba G. Blazquez*[1,2], Rocio I.R. Macias*[1,2], Candela Cives-Losada[1], Alberto de la Iglesia[1], Jose J.G. Marin**[1,2], Maria J. Monte**[1,2]

1) Experimental Hepatology and Drug Targeting (HEVEFARM), IBSAL, University of Salamanca, Salamanca, Spain.

2) Center for the Study of Liver and Gastrointestinal Diseases (CIBERehd), Carlos III National Institute of Health, Madrid, Spain.

Supplementary Table 1. Oligonucleotide sequence of primers used to determine by RT-QPCR the expression levels of rat ABC transporters.

Protein	Gene	Forward Primer (5´-3´)	Reverse Primer (5´-3´)	Amplicon Size	Accession
Bcrp	*Abcg2*	ATGATGCTCTTTTCTGGCCTCT	AAGCCATATCGAGGAATGCTAAA	92	NM_181381
Bsep	*Abcb11*	GCCATTGTGCGAGATCCTAAA	TGCAGGTCCGACCCTCTCT	118	NM_031760
Mrp1	*Abcc1*	ATCCGCTCTGGGATTGGAAT	AGAGGTAGAAACAAGGCACCCA	102	NM_022281
Mrp2	*Abcc2*	TGATCGGTTTGCTGAAGAGCT	ACGCACATTCCCAACACAAA	139	NM_012833
Mrp3	*Abcc3*	AAGGAGTCTTGCAACATGCAAAT	CAAGGAGCTCATCTCTCTCATGAA	134	NM_080581
Mrp4	*Abcc4*	TGTCAATCATACCTCAGGAACCTGT	TGTACCTCCTCTAGGGCTTTCCA	112	NM_133411
Cyp7a1	*Cyp7a1*	GCTTTACAGAGTGCTGGCCAA	CTGTCTAGTACCGGCAGGTCATT	92	NM_012942
Cyp27a1	*Cyp27a1*	CCTTTGGGACTCGCACCA	GCCCTCCTGTCTCATCACTTG	70	NM_73231
Fxr	*Nr1h4*	GAAAGAGCGGCATCTCCGA	TGCTGTGAGCAGAGCGTACTCTT	104	NM_021745
Shp	*Nr0b2*	TCTTCAACCCAGATGTGCCAG	GAAGCCATGAGGAGGATTCG	145	NM_057133
β-Actin	*Actb*	GCCACCAGTTCGCCATGGAT	CCCACGATGGAGGGGAAGA	121	NM_031144

Supplementary Table 2. Oligonucleotide sequence of primers used to determine by RT-QPCR the expression levels of mice ABC transporters.

Protein	Gene	Forward Primer (5´-3´)	Reverse Primer (5´-3´)	Amplicon Size	Accession
Bcrp	*Abcg2*	GCAGCAAGGAAAGATCCAAAGGGATTA	CCATCACAACGTCATCTTGAACCACA	114	NM_011920
Bsep	*Abcb11*	TGACTTTCCACAGTGGCGTCT	ATGGTGTCTGCAATCTTCACTCA	87	NM_0021022
Mrp1	*Abcc1*	GTCATGAGGGCGGCAAGAT	CCCTGACCACTGACACTGTCA	144	NM_008576
Mrp2	*Abcc2*	TCTAGAGACGGATAGCTCATTCA	CGCTGTCTAGGACCATTACCTTGT	125	NM_013806
Mrp3	*Abcc3*	CTCTCAGCTTTGGCAGAGGGTAAAATCTT	CCTCTGGGCAAGGATTTGTGTCAA	160	NM_029600
Mrp4	*Abcc4*	GCGTGTTCTTCTGGTGGCTCA	GCAGAAGTTCTTTATCCCAGTACCGTT	94	NM_001033336
β-Actin	*Actb*	GCCACCAGTTCGCCATGGAT	ACCATCACACCCTGGTGCCTA	148	NM_007393

Supplementary Table 3. Relative mRNA levels of bile acid homeostasis-related genes in the liver of 10-days old rats.

**Actual Mother**		**Control**	**BDL**	**Control**	**BDL**
**Breastfeeding Mother**		**Control**	**BDL**	**BDL**	**Control**
Protein	*Gene*	*mRNA Abundance (% of maternal control liver)*			
Cyp7a1	*Cyp7a1*	51.6±4.6	61.5±20.4	61.7±8.5	32.4±10.2
Cyp27a1	*Cyp27a1*	130±15	168±19	106±15	129±19
Fxr	*Nr1h4*	43.1±3.1	40.8±5.3	46.8±4.0	43.1±3.0
Shp	*Nr0b2*	586±106	655±103	529±63	518±87

Steady-state levels of mRNA in liver tissue obtained from the offspring of healthy control and cholestatic (BDL) lactating rats on day 10 after delivery. Obstructive cholestasis was imposed on the mothers by bile-duct ligation on day 14 of pregnancy. In two additional groups litters and mothers were exchanged immediately after delivery in order to impose breastfeeding of pups born from control rats by BDL rats and pups born from BDL rats by control rats. Values are mean±SEM from measurements by RT-QPCR of samples from 9-15 pups belonging to 3 to 5 different litters in each case, normalized by the abundance of mRNA of β-actin in each sample and expressed as percentage of the expression on maternal control liver. No significant differences were found among the groups by the Bonferroni method of multiple-range testing.
